# Screening Biomarkers for Systemic Lupus Erythematosus Based on Machine Learning and Exploring Their Expression Correlations With the Ratios of Various Immune Cells

**DOI:** 10.3389/fimmu.2022.873787

**Published:** 2022-06-10

**Authors:** Yafang Zhong, Wei Zhang, Xiaoping Hong, Zhipeng Zeng, Yumei Chen, Shengyou Liao, Wanxia Cai, Yong Xu, Gang Wang, Dongzhou Liu, Donge Tang, Yong Dai

**Affiliations:** ^1^Clinical Medical Research Center, Guangdong Provincial Engineering Research Center of Autoimmune Disease Precision Medicine, Shenzhen Engineering Research Center of Autoimmune Disease, The Second Clinical Medical College of Jinan University, Shenzhen People’s Hospital, Shenzhen, China; ^2^South China Hospital, Health Science Center, Shenzhen University, Shenzhen, China; ^3^The First Affiliated Hospital of Shenzhen University, Shenzhen Second People’s Hospital, Shenzhen, China; ^4^Department of Nephrology, Shenzhen Hospital, University of Chinese Academy of Sciences, Shenzhen Guangming New District Hospital, Shenzhen, China

**Keywords:** machine learning, diagnostic biomarker, systemic lupus erythematosus, immune cell disturbance, CIBERSORT

## Abstract

**Background:**

Systemic lupus erythematosus (SLE) is an autoimmune illness caused by a malfunctioning immunomodulatory system. China has the second highest prevalence of SLE in the world, from 0.03% to 0.07%. SLE is diagnosed using a combination of immunological markers, clinical symptoms, and even invasive biopsy. As a result, genetic diagnostic biomarkers for SLE diagnosis are desperately needed.

**Method:**

From the Gene Expression Omnibus (GEO) database, we downloaded three array data sets of SLE patients’ and healthy people’s peripheral blood mononuclear cells (PBMC) (GSE65391, GSE121239 and GSE61635) as the discovery metadata (n_SLE_ = 1315, n_normal_ = 122), and pooled four data sets (GSE4588, GSE50772, GSE99967, and GSE24706) as the validate data set (n_SLE_ = 146, n_normal_ = 76). We screened the differentially expressed genes (DEGs) between the SLE and control samples, and employed the least absolute shrinkage and selection operator (LASSO) regression, and support vector machine recursive feature elimination (SVM-RFE) analyze to discover possible diagnostic biomarkers. The candidate markers’ diagnostic efficacy was assessed using the receiver operating characteristic (ROC) curve. The reverse transcription quantitative polymerase chain reaction (RT-qPCR) was utilized to confirm the expression of the putative biomarkers using our own Chinese cohort (n_SLE_ = 13, n_normal_ = 10). Finally, the proportion of 22 immune cells in SLE patients was determined using the CIBERSORT algorithm, and the correlations between the biomarkers’ expression and immune cell ratios were also investigated.

**Results:**

We obtained a total of 284 DEGs and uncovered that they were largely involved in several immune relevant pathways, such as type І interferon signaling pathway, defense response to virus, and inflammatory response. Following that, six candidate diagnostic biomarkers for SLE were selected, namely ABCB1, EIF2AK2, HERC6, ID3, IFI27, and PLSCR1, whose expression levels were validated by the discovery and validation cohort data sets. As a signature, the area under curve (AUC) values of these six genes reached to 0.96 and 0.913, respectively, in the discovery and validation data sets. After that, we checked to see if the expression of ABCB1, IFI27, and PLSCR1 in our own Chinese cohort matched that of the discovery and validation sets. Subsequently, we revealed the potentially disturbed immune cell types in SLE patients using the CIBERSORT analysis, and uncovered the most relevant immune cells with the expression of ABCB1, IFI27, and PLSCR1.

**Conclusion:**

Our study identified ABCB1, IFI27, and PLSCR1 as potential diagnostic genes for Chinese SLE patients, and uncovered their most relevant immune cells. The findings in this paper provide possible biomarkers for diagnosing Chinese SLE patients.

## Introduction

Systemic lupus erythematosus (SLE) is a multisystem autoimmune disease that primarily affects the adolescent and menopausal women (1). The clinical manifestations of SLE are heterogeneous, involving the blood, kidney, nerve and some other systems, making the treatment and management difficult and complicated (2). Currently, the Chinese hospitals often adopt European and American (EULAR/ACR) diagnostic guidelines for SLE diagnosis (3, 4), whereas the molecular profiling of SLE is highly heterogeneous among different races and locations (5, 6). Consequently, the specificity and sensitivity of diagnostic indicators recommended by the EULAR/ACR need to be validated further in Chinese population.

Nowadays, the diagnosis of SLE is primarily based on a series of clinical manifestations and laboratory indicators, such as skin erythema, arthralgia, complement C3, C4, and anti-dsDNA antibodies, etc. (7–11). SLE must be diagnosed based on clinical signs along with multiple immunological indicators. When patients were diagnosed as SLE, they often had occurred a certain degree of systemic involvements (12). Even an invasive biopsy is required to diagnose lupus nephritis (13). Because of the complexity, lag, and invasiveness of current SLE diagnostic methods, researchers are looking for new genetic biomarkers in the hopes of developing a simpler, faster, and more objective diagnostic “gold standard” than current markers/tests, without the need for clinical symptoms, especially in the Chinese population.

Until now, immune cells such as lymphocytes, dendritic cells, macrophages, basophils, and neutrophils have been identified to be disrupted in the course of SLE (14–17). There are differences in the disturbed cell types and proportions between SLE individuals (18–21). The identification of altered immune cells in SLE aids us in understanding the cellular results of the disease and establishing an appropriate diagnostic or treatment strategy.

In this study, we used the Gene Expression Omnibus (GEO) database to obtain three expression microarray datasets of SLE patients’ and healthy people’s peripheral blood mononuclear cells (PBMC). Then, we pooled the three datasets as a metadata cohort (n_SLE_ = 1315, n_normal_ = 122) and looked for the differentially expressed genes (DEGs) between SLE and controls. Next, we identified the diagnostic biomarkers of SLE using different machine learning algorithms. Following that, we merged four GEO data sets as the validation data (GSE4588, GSE50772, GSE99967, and GSE24706; n_SLE_ = 146, n_normal_ = 76) and confirmed the expression of the identified diagnostic biomarkers, then used logistic regression to develop a diagnostic prediction model. Furthermore, we validated the expression of the candidate biomarkers using our own Chinese cohort (n_SLE_ = 13, n_normal_ = 10). Moreover, we used the CIBERSORT algorithm to quantify the proportion of 22 immune cells in PBMC of SLE patients and healthy people. Finally, we explored the relationship between the expression of the identified biomarkers and the ratios of immune cells in PBMC of SLE patients.

## Materials and Methods

### Data Download and Processing

Seven expression matrix files of SLE PBMC samples were downloaded from the GEO database, namely GSE65391, GSE121239, GSE61635, GSE4588, GSE50772, GSE99967, and GSE24706. The GSE65391 dataset contained 924 SLE and 72 control samples, the GSE121239 dataset contained 292 SLE and 20 control samples, GSE61635 dataset contained 99 SLE and 30 control samples, the GSE4588 dataset contained 15 SLE and 19 control samples, the GSE50772 dataset contained 61 SLE and 20 control samples, the GSE99967 dataset contained 42 SLE and 17 control samples, and GSE24706 dataset contained 28 SLE and 20 control samples. Then, the three files GSE65391, GSE121239, and GSE61635 were pooled into a metadata cohort for the following analysis, after the batch effects were removed using the R package “SVA”. In addition, the four data sets (GSE4588, GSE50772, GSE99967, and GSE24706) were also merged as the verification data after normalization.

### DEGs Screening and Functional Analysis

The DEGs were screened out using the R package “limma” based on the metadata cohort’s data set. The heat map showing the expression of DEGs was depicted using the R package. Next, the DAVID database was used to analysis the functions of the DEGs based on gene ontology (GO) and Kyoto Encyclopedia of Genes and Genomes (KEGG) pathway. In addition, the R package “clusterProfiler” and “DOSE” were used for the Disease ontology (DO) enrichment analyses. The gene set enrichment analysis (GSEA) was used to distinguish the most significant functional items between SLE and controls. The gene set “c2.cp.kegg.v7.0.symbols.gmt” from the Molecular Signatures Database (MSigDB) (22) was selected as the reference gene set.

### Identification and Verification of the Diagnostic Markers

Two algorithms, namely least absolute shrinkage and selection operator (LASSO) logistic regression and support vector machine-recursive feature elimination (SVM-RFE), were used to screen the potential SLE diagnostic markers. The “glmnet” R package was used to perform the LASSO analysis to identify the optimal diagnostic markers in SLE, and the SVM-RFE was used to find the optimal variables. The candidate diagnostic markers were picked by intersecting the markers identified by these two algorithms. Then, the expression of the candidate diagnostic biomarkers were verified based on the merged dataset containing GSE4588, GSE50772, GSE99967, and GSE24706.

### The Diagnostic Efficacy of the Biomarkers in SLE

To examine the diagnostic efficacy of the candidate markers, the receiver operating characteristic (ROC) curves were drawn based on the discovery metadata and the validation data set.

### Patients

A total of 23 whole blood samples (including 13 SLE samples and 10 control samples) were collected from Shenzhen People’s Hospital. Patients diagnosed as SLE, with a SLE disease activity index (SLEDAI) score more than 5 were included. The clinical manifestations of all patients were shown in **Table 1**. All participants volunteered to participate in this research. This work was approved by the Ethics Committee of Shenzhen People’s Hospital (LL-KY-2021393). After collecting all of the whole blood samples, the PBMCs were isolated, and dissolved in Trizol (Beyotime, R-0016), and then stored in -80 °C.

**Table 1:** The clinical information of the 13 SLE patients and 10 healthy people in our study.

	SLE (n = 13)	Healthy people (n = 10)
**Gender (female/male)**	11/2	9/1
**Age (year), median (range)**	38 (15-57)	31 (26-37)
**C3(g/L), median (range)**	0.76 (0.21-0.85)	N/A
**C4(g/L), median (range)**	0.14 (0.03-0.28)	N/A
**ANA(+) (percentage)**	13 (100%)	N/A
**Anti-dsDNA(+) (percentage)**	10 (76.9%)	N/A
**ANuA(+) (percentage)**	9 (69.2%)	N/A
**Proteinuria (g/24 h), median (range)**	0.363 (0.045-10.98)	N/A
**SLEDAI-2K, median (range)**	9 (6-21)	N/A
**Rash (percentage)**	3 (23.1%)	N/A
**Alopecia (percentage)**	2 (15.4%)	N/A
**Fever (percentage)**	4 (30.8%)	N/A
**Pleurisy (percentage)**	3 (23.1%)	N/A
**Leukopenia (percentage)**	4 (30.8%)	N/A
**Pericarditis (percentage)**	3 (23.1%)	N/A
**Arthritis (percentage)**	2 (15.4%)	N/A
**Organic encephalopathy syndrome (percentage)**	1 (7.7%)	N/A
**Hematuria (percentage)**	1 (7.7%)	N/A
**Thrombocytopenia (percentage)**	2 (15.4%)	N/A
**Lupus headache (percentage)**	1 (7.7%)	N/A
**Vasculitis (percentage)**	2 (15.4%)	N/A

SLE, systemic lupus erythematosus; ANA, anti-nuclear antibody; Anti-dsDNA, anti-dsDNA antibodies; ANuA, anti-nucleosome antibodies; C3, Complement C3; C4, Complement C4; N/A, not applicable; SLEDAI-2K, SLE disease activity index 2000.

### Reverse Transcription Quantitative Polymerase Chain Reaction (RT-qPCR)

The total RNA was isolated from the PBMC according to the manufacturer’s instructions. The transScript all-in-one first-strand cDNA synthesis superMix for qPCR (One-step gDNA removal) kit (TransGen Biotech, AT341-01) was used for the reverse transcription of mRNA. After that, the qPCR assays were conducted out with the PerfectStart Green qPCR SuperMix kit (TransGen Biotech, AQ601-02), with the following primers: GAPDH (Forward: 5-TGACTTCAACAGCGACACCCA-3. Reverse: 5-CACCCTGTTGCTGTAGCCAAA-3). ABCB1 (Forward: 5-GTCTGGACAAGCACTGAAAGATAAGA-3. Reverse: 5- CAACGGTTCGGAAGTTTTCTATTGC-3). EIF2AK2 (Forward: 5- AGAAGGCGGAGCGTGAAGTAAAAG-3. Reverse: 5- ATCCATCCCAACAGCCATTGTAGTG-3). HERC6 (Forward: 5- CCTGCCAAGCCTAAACCTGAGAAG-3. Reverse: 5- TACAGAGCCAGTGGGAAAGGAAGG-3). ID3 (Forward: 5-GGACGACATGAACCACTGCT-3. Reverse: 5-TCAGTGGCAAAAGCTCCTTT-3). IFI27 (Forward: 5-TCTGCAGTCACTGGGAGCAA-3. Reverse: 5-CCCAATGGAGCCCAGGAT-3), PLSCR1 (Forward: 5-CCTCAGTATCCACCGACAGCATTC-3. Reverse: 5-ACTGGCTGATTTGGGACAGGAAAG-3). All the primers were synthesized by the Sangon Biotech Company. Among them, GAPDH was an internal reference gene. The gene relative expressions were calculated by the 2-ΔΔCT method. A p value < 0.05 was considered statistically significant.

### Immune Cell Composition

The CIBERSORT algorithm (23) was used to calculate the ratios of various immune cells in PBMC of SLE patients and healthy people based on the expression matrix, and the R package “vioplot” was used to visualize the proportions of 22 immune cells in SLE and control groups. The “corrplot” package was used to create the heat map displaying the quantitative correlation between distinct immune cells. In addition, the “ggplot2” R package was utilized to investigate the association between the expression of the diagnostic markers and the ratios of immune cells.

## Results

### Screening the DEGs Between the SLE PBMC Versus Control PBMC

**Figure 1:**
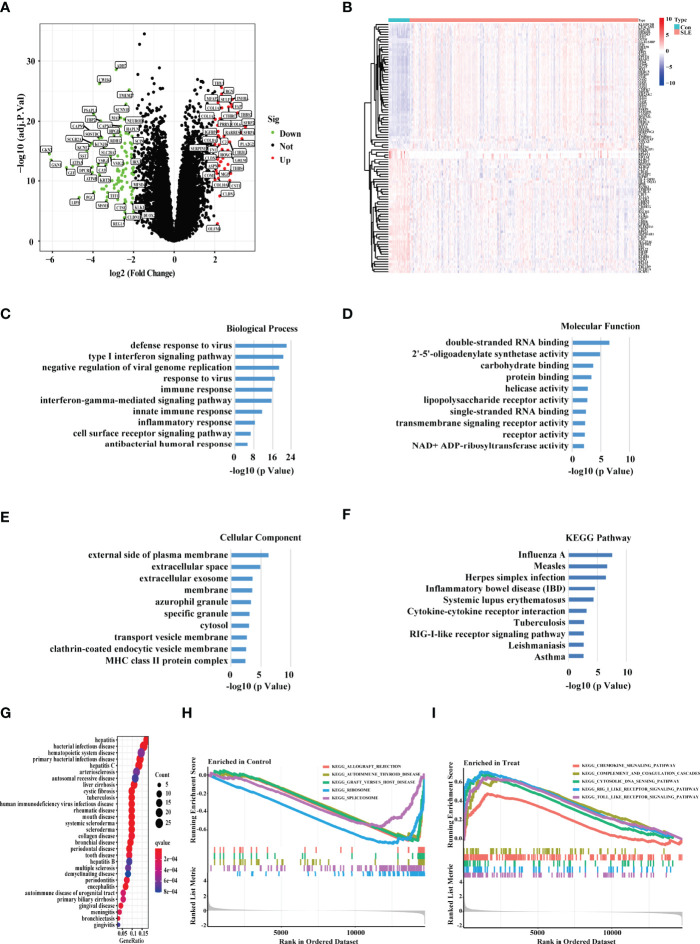
Screening the DEGs between the SLE PBMC versus Control PBMC. **(A)** Volcano plot showing the DEGs between the PBMC of SLE and control samples based on the metadata set including GSE65391, GSE121239, and GSE61635. Green represented down-regulation, and red represented up-regulation (SLE/Control). **(B)** The heat map showing the DEGs in each sample of SLE patients and healthy people. “Con” represented control samples, and “SLE” represented SLE patients. **(C–E)** The GO, **(F)** KEGG, and **(G)** DO enrichment analysis of the DEGs. The size of the dots represented the number of genes in each enriched module. The color of the dots represented the q value. **(H–I)** The GSEA analysis revealing the enriched pathways in the PBMC of SLE and control samples.

After batch effects were removed using the R package “SVA,” we combined three expression array data sets collected from the GEO database (GSE65391, GSE121239, and GSE61635) into one discovery data set, which included data from 1315 SLE patients and 122 healthy people. We used principal component analysis (PCA) to cluster all of the samples and discovered that each sample’s point was randomly distributed, indicating that the normalization was done correctly (**Supplementary Figure S1**). As a consequence, 284 genes were discovered to be differentially expressed in the PBMC of SLE patients versus healthy people (p value < 0.0001, fold change > 1.5) (**Figure 1A**). We used the heat map showing the expression of the DEGs in each healthy people and SLE patient (**Figure 1B**). Through an enrichment analysis of the DEGs, we found that these DEGs were enriched in some typical autoimmune-disease-relevant pathways, such as type І interferon signaling pathway, innate immune response, inflammatory response, and systemic lupus erythematosus (**Figures 1C–F**). In addition, we conducted the DO enrichment analyses of the DEGs, and discovered that the DEGs were primarily involved in several immune related diseases, namely bacterial infectious disease, hematopoietic system disease, human immunodeficiency virus infectious disease, and rheumatic disease, etc. (**Figure 1G**). Furthermore, the GSEA results showed that the DEGs in SLE patients were mainly involved with chemokine signaling pathway, complement and coagulation cascades, cytoplasmic DNA sensing pathway, RIG-I-like receptor signaling pathway, and Toll-like receptor signaling pathway (**Figures 1H, I**).

### Identification of the Potential Diagnostic Biomarkers for SLE Based on Machine Learning

**Figure 2:**
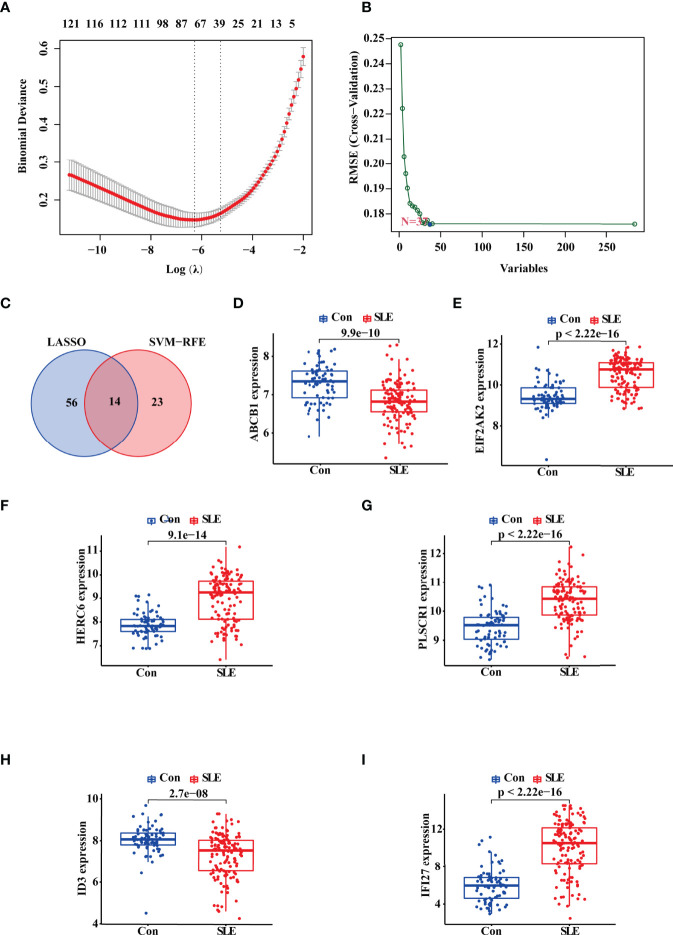
Identification and Validation of the Diagnostic Biomarkers for SLE. **(A, B)** LASSO logistic regression and SVM-RFE algorithm screening diagnostic biomarkers for SLE. **(C)** The Venn diagram showing the intersection of the diagnostic biomarkers screened by the two algorithms. **(D–I)** Validation of the expression of diagnostic biomarkers based on the validate data set including GSE4588, GSE50772, GSE99967, and GSE24706. “Con” represented the control samples, and “SLE” represented the SLE patients.

Then, we adopted two machine learning algorithms to screen the biomarkers for SLE, namely LASSO regression and SVM-RFE algorithm. As a result, the LASSO regression algorithm revealed 70 probable biomarkers, while the SVM-RFE approach identified 37 (**Figures 2A, B**). After that, we made an intersection of the 70 and 37 probable biomarkers to arrive at 14 common biomarkers (**Figure 2C**; **Supplementary Table S1**). To further confirm the reliability of these biomarkers, we verified their expressions based on the validation data set (n_SLE_ = 146, n_normal_ = 76). The result showed that 10 genes harbored the similar expression trend with statistical significance in both discovery and validation data sets, including ABCB1, EIF2AK2, HERC6, PLSCR1, ID3, IFI27, SCRN1, CD160, HSP90AB1, and PCYOX1. Among these 10 genes, we selected ABCB1, EIF2AK2, HERC6, PLSCR1, ID3, and IFI27 for the following study because the statistical differences of their expression was most significant in both the discovery and validation sets (**Figures 2D–I**).

### The Diagnostic Efficacy of the Six Candidate Biomarkers for SLE

**Figure 3:**
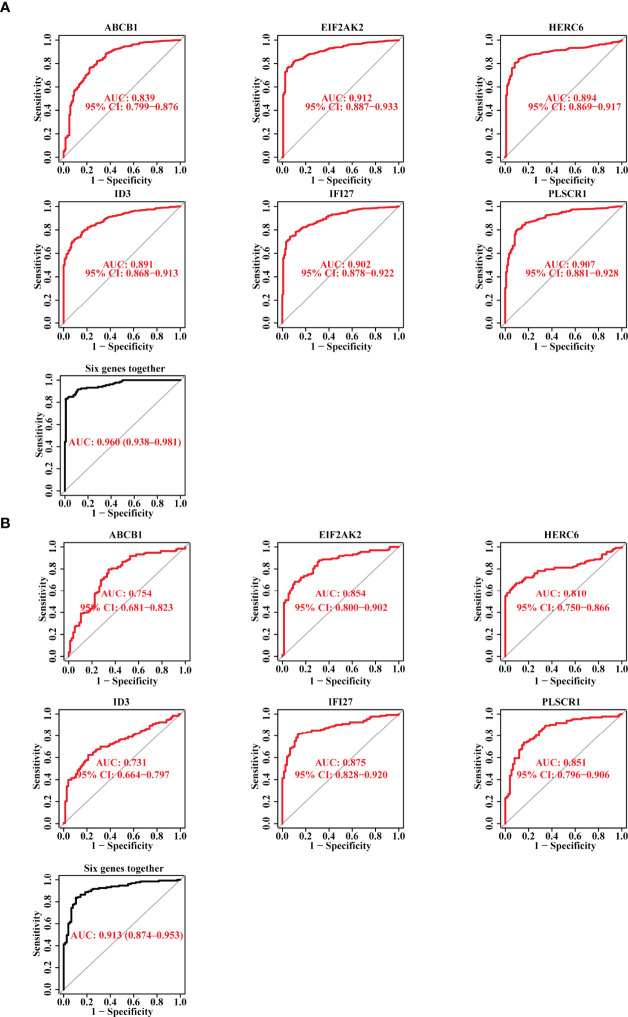
The Diagnostic Efficacy of the Selected Diagnostic Markers for SLE. **(A)** The ROC curve showing the AUC value of ABCB1, EIF2AK2, HERC6, ID3, IFI27, and PLSCR1 based on the data set of the discovery cohort. **(B)** The ROC curve showing the AUC value of ABCB1, EIF2AK2, HERC6, ID3, IFI27, and PLSCR1 based on the data set of the validation cohort.

Subsequently, we plotted the ROC curves for the six candidate biomarkers, and found that ABCB1, EIF2AK2, HERC6, ID3, IFI27, and PLSCR1 all had a good diagnostic effect in the discovery data set, with an AUC value 0.839, 0.912, 0.894, 0.891, 0.902, and 0.907, respectively. When we used the six markers as one single signature, the AUC value reached 0.96 (**Figure 3A**). On the other hand, we also verified the diagnostic effect of the six genes in the validation data set, and discovered that the AUC values of the six biomarkers, including ABCB1, EIF2AK2, HERC6, ID3, IFI27, and PLSCR1, were 0.754, 0.854, 0.81, 0.731, 0.875, and 0.851, respectively. When the six markers were combined as one single signature, the AUC value reached 0.913 (**Figure 3B**). The result suggests that the six genes have a good diagnostic power for SLE, and the power is higher when they are used together.

### The Validation of the Potential Diagnostic Markers Using Our Own Chinese Cohort Revealed that ABCB1, IFI27 and PLSCR1 Were More Likely to be SLE Biomarkers for the Chinese Population

**Figure 4:**
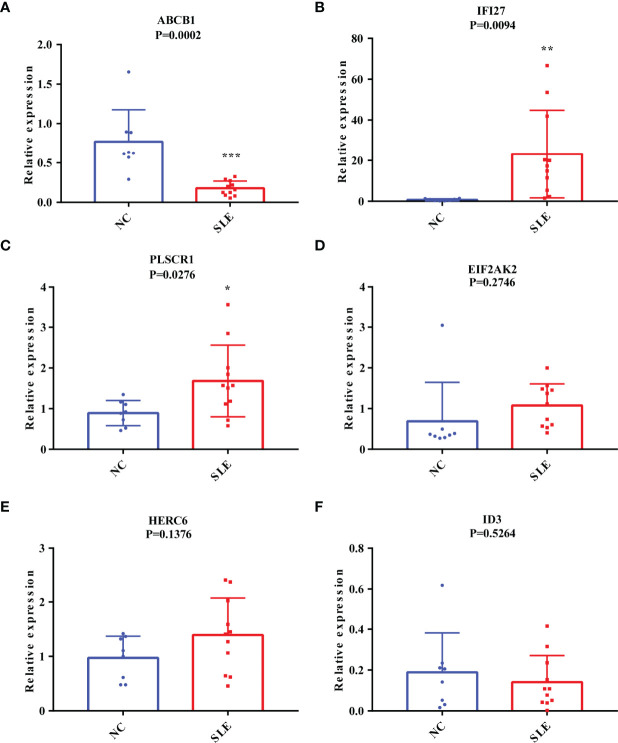
The Validation of the Putative Diagnostic Markers by RT-qPCR in Chinese population using Our Own Cohort. **(A-F)** The relative mRNA expression levels of ABCB1, IFI27, PLSCR1, EIF2AK2, HERC6, and ID3 in the PBMC of SLE patients and healthy people. “*” represented P <0.05, “**” represented P <0.01, and “***” represented P <0.001.

As mentioned above, SLE is highly heterogeneous among different races and regions (24). To ensure that the biomarkers we screened could be applied in the Chinese population, we further performed the RT-qPCR assays on the expression of these six markers in the PBMC of 13 Chinese SLE patients and 10 Chinese healthy controls (**Supplementary Table S1**). Consistently, the mRNA expression level of ABCB1 in PBMC of SLE patients was significantly lower than that of healthy controls (**Figure 4A**). SLE patients had considerably greater levels of IFI27 and PLSCR1 expression than healthy controls (**Figures 4B, C**). Meanwhile, we found that the expression differences of EIF2AK2, HERC6 and ID3 in SLE patients and healthy controls followed the same pattern as the discovery and validation cohorts (though the difference was not statistically significant) (**Figures 4D–F**). Thus, these results indicate that our findings are reproducible, and ABCB1, IFI27 and PLSCR1 are more likely to be SLE biomarkers in the Chinese population. Notably, ABCB1 is a novel found biomarker for SLE that has not yet to be published, to the best of our knowledge.

### The Ratio Changes of Immune Cells in SLE Patients, and Their Correlation With the Expression of ABCB1, IFI27 and PLSCR1

**Figure 5:**
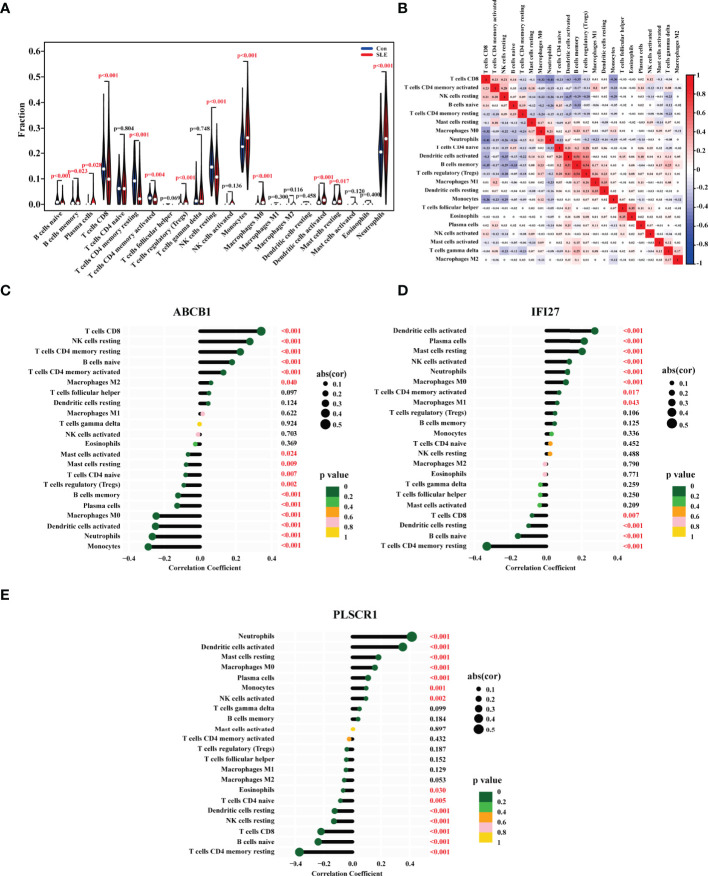
The Ratios of Immune Cells in the PBMC of SLE Patients and their Correlations with the Biomarkers’ Expression. **(A)** Violin diagram showing the proportion of 22 types of immune cells in the PBMC of SLE patients and healthy people. The red marks represented p < 0.05. **(B)** The heat map showing the proportion correlation between the 22 types of immune cells in SLE patients. The size of the colored squares represented the strength of the correlation, and red represented a positive correlation, and blue represented a negative correlation. The correlation between **(C)** ABCB1, **(D)** IFI27, **(E)** PLSCR1 expression with the degrees of immune cells in SLE. The size of the dots represented the strength of the correlation. The color of the dots represented the p value. A p < 0.05 was considered statistically significant.

The onset of SLE causes changes in the proportion and function of a series of immune cells. To find the biomarkers whose expression were correlated with the proportions of immune cells, we first analyzed the ratio changes of 22 immune cells in 1315 SLE patients and 122 healthy people using the CIBERSORT algorithm. The results showed that compared with the control group, the proportions of naive B cells, CD8 T cells, CD4 memory resting T cells, CD4 memory activated T cells, and resting natural killer (NK) cells in SLE were significantly lower, while monocytes, macrophages M0, activated dendritic cells, neutrophils were higher (**Figure 5A**). Consistently, a number of previous studies have also demonstrated that the ratio of resting NK cells was decreased in SLE patients and the ratios of monocytes, neutrophils were increased in SLE patients (25–27).

Following that, we investigated the correlation between the ratios of the 22 types of immune cells in SLE patients, and discovered that the degrees of memory B cells and activated dendritic cells, the levels of regulatory T cells (Tregs) and memory B cells, the levels of Tregs and activated dendritic cells all had a strong positive link, respectively. Furthermore, the ratio of CD8 T cells was adversely linked with that of neutrophils, memory B cells, and monocytes (**Figure 5B**).

Finally, we looked at the relationship between the immune cell ratios and the expression of ABCB1, IFI27 and PLSCR1 in SLE patients. As a result, the ABCB1 expression was positively correlated with the levels of CD8 T cells, resting NK cells, CD4 memory resting T cells, naive B cells, CD4 memory activated T cells, and macrophages M2, and negatively correlated with the ratios of activated mast cells, resting mast cells, CD4 naive T cells, Tregs, memory B cells, plasma cells, macrophages M0, activated dendritic cells, neutrophils, and monocytes (**Figure 5C**). The IFI27 expression was positively linked with the degrees of activated dendritic cells, plasma cells, resting mast cells, activated NK cells, neutrophils, macrophages M0, memory activated cells CD4, and macrophages M1, and negatively linked with CD8 T cells, resting dendritic cells, naive B cells, and CD4 memory resting T cells (**Figure 5D**). The PLSCR1 expression was positively correlated with the ratios of neutrophils, activated dendritic cells, resting mast cells, macrophages M0, plasma cells, monocytes, and activated NK cells, and negatively correlated with eosinophils, CD4 naive T cells, resting dendritic cells, resting NK cells, CD8 T cells, naive B cells, CD4 memory resting T cells (**Figure 5E**). All in all, the ABCB1 expression was most closely linked to the ratios of CD8 T cells and monocytes, the IFI27 expression to the levels of activated dendritic cells and CD4 memory resting T cells, and the PLSCR1 expression to the degrees of neutrophils and CD4 memory resting T cells.

## Discussion

SLE is a type of multi-system autoimmune disease, the pathophysiological mechanisms of which have not been thoroughly elucidated (27). In clinic, SLE is diagnosed according to the clinical features, such as injures of skin, joints, kidneys, nervous system, as well as the serologic parameters, such as autoimmune antibody. Furthermore, the diagnosis of SLE must be based on the clinical manifestations (such as organ damages) together with multiple immunological indicators (12, 28). Some types of organ damage need to be diagnosed in an invasive way, causing great pain to the patients. Therefore, the diagnosis of SLE is a tedious, complex and time-consuming process, and it is of great significance to search for genetic markers for SLE diagnosis.

In this study, we screened the putative diagnostic biomarkers of SLE using machine learning. We all know that the conclusions drawn from a single data set are typically limited, unrepeatable, or inconsistent. To ensure the correctness of our findings, we retrieved three cohorts from the GEO database (n_SLE_ = 1315, n_normal_ =122). Among the six putative biomarkers, we confirmed their expression using our own Chinese cohort (n_SLE_ =13, n_normal_ =10), and three of them were statistically significant, while the other three genes showed the same expression trend to the machine learning results. The reason for this phenomenon could be due to the small sample size and the racial heterogeneity. Thus, a bigger cohort may be necessary for the further validation in future research.

In 2021, Zhao X’s team has revealed several potential SLE biomarkers based on a comprehensive bioinformatics analysis (27). Compared with this paper, we have the following advancements, extensions and deeper studies: First, we established a larger discovery cohort (n_SLE_ = 1315, n_normal_ =122) to ensure that the markers screened out are more universal and accurate. Second, we used two machine learning methods, LASSO and SVM, to screen diagnostic markers, whereas Zhao X’s work screened biomarkers through overlapping the DEGs of six independent data sets. Compared with the intersection method, LASSO and SVM have stronger power in identifying biomarkers with higher distinguishing efficacy. Third, we validated the screened markers using our own Chinese cohort, thereby the markers had potential application for Chinese patients.

We also observed a link between the infiltration of certain immune cells and the expression of biomarker genes. However, more evidence is needed to support the link in a wider range of situations, and the relevance of this correlation is unknown at this time. Furthermore, immune cell infiltration and marker gene expression may not be causally related. The systemic inflammation that occurs in lupus may cause changes in immune cell ratio, and the marker gene expression may be linked to that inflammation.

Among the six SLE candidate markers, ABCB1, HERC6 and ID3 are identified for the first time. Furthermore, we confirmed the expression of ABCB1 in the Chinese population using our own cohort. ABCB1, also known as P-glycoprotein (P-GP), is a multidrug resistance protein mainly expressing in barrier organs such as brain, liver, kidney, and skin (29–32). Recently, ABCB1 has been reported being expressed in a variety of immune cells, such as monocytes, antigen presenting dendritic cells, T cells and B cells, and is involved in the efflux of inflammatory molecules (33). Therefore, it’s possible that ABCB1 plays a role in SLE by participating in the inflammatory pathways. Besides, HERC6 has been revealed as a key determinant of cellular antiviral activity, and is also one constituent gene of type І interferon signaling pathway (34, 35), which was regarded as a central pathogenic pathway in SLE (36). Additionally, several studies have proclaimed that ID3 is a key regulator of IL-5 production and the homeostasis of B-1a B cells (37). ID3 helps the tuberculosis subunit vaccine to induce long-term immune memory, providing immune protection against M tuberculosis infection (38).

Machine learning, on the other hand, detected three recognized SLE biomarkers, including EIF2AK2, IFI27, and PLSCR1, confirming the algorithms’ accuracy. The protein encoded by EIF2AK2, as one key component of the innate immune system, is increasingly expressed in T cells of SLE patients, and is likely to be associated with cellular translation and proliferation (39). Besides, EIF2AK2 selectively regulates the transcription of genes functioning in immune response in SLE (39). In proteasome-associated autoinflammatory cell models, the activation of EIF2AK2 is discovered responding to the decreased proteasome function (40). Numerous studies have demonstrated the indispensable role of IFI27 (Interferon (IFN)-α-inducible protein 27) in SLE (41–43). IFI27 is strongly correlated with the levels of T helper type 1 (Th1) cells, T helper type 2 (Th2) cells and activated dendritic cells (aDC), and the up-regulation of IFI27 is highly related to many inflammatory events induced by these immune cells (44, 45). Ultimately, PLSCR1 has been found an increased expression in multiple systemic autoimmune diseases, such as primary antiphospholipid syndrome, rheumatoid arthritis, idiopathic inflammatory myopathies and SLE (46, 47). Meanwhile, the PLSCR1 expression was discovered to be associated with type I interferon-stimulated genes (48), and was higher in neutrophils, dendritic cells, and macrophages (43), which is consistent with our results.

## Conclusions

Generally speaking, we identified six genes as prospective SLE biomarkers (n_SLE_ = 1315, n_normal_ =122), including ABCB1, EIF2AK2, HERC6, ID3, IFI27, and PLSCR1, and demonstrated that ABCB1, EIFI27, and PLSCR1 might be suitable for the Chinese population. Meanwhile, we discovered the quantitative changes of 13 types of immune cells in SLE patients, as well as the link between the expression of ABCB1, IFI27, and PLSCR1 and the ratios of different immune cells. Our findings provide potential biomarkers for Chinese SLE patients, and give the insight into the relationship between gene expression and immune cell ratios.

## Data Availability Statement

The datasets presented in this study can be found in online repositories. The names of the repository/repositories and accession number(s) can be found in the article/**Supplementary Material**.

## Ethics Statement

The studies involving human participants were reviewed and approved by the Ethics Committee of Shenzhen People’s Hospital (LL-KY-2021393). Written informed consent to participate in this study was provided by the participants’ legal guardian/next of kin.

## Author Contributions

WZ conceived and designed the project. YZ wrote the manuscript. SL provided data analysis support. XH and DL contributed to clinical sample collection. YC, ZZ, and WC performed the experiments. YX and GW provided conceptual guidance. DT and YD supervised the study. All authors read, revised and approved the final manuscript.

## Funding

This study was supported by grants from the Key Research and Development Program of Guangdong Province (No. 2019B020229001), the science and technology plan of Shenzhen (No. JCYJ20200109144218597), Shenzhen Guangming District Science and Technology Innovation Bureau (No. 2021R01020), Shenzhen Key Medical Discipline Construction Fund (No. SZXK011), and the Guangdong Basic and Applied Basic Research Foundation (No. 2021A1515111071).

## Conflict of Interest

The authors declare that the research was conducted in the absence of any commercial or financial relationships that could be construed as a potential conflict of interest.

## Publisher’s Note

All claims expressed in this article are solely those of the authors and do not necessarily represent those of their affiliated organizations, or those of the publisher, the editors and the reviewers. Any product that may be evaluated in this article, or claim that may be made by its manufacturer, is not guaranteed or endorsed by the publisher.
