# Supplementary figures and images for: Screening Biomarkers for Systemic Lupus Erythematosus Based on Machine Learning and Exploring Their Expression Correlations With the Ratios of Various Immune Cells
Source: Front Immunol. 2022 Jun 10;13:873787. doi: 10.3389/fimmu.2022.873787 (PMC9226453; doi:10.3389/fimmu.2022.873787)

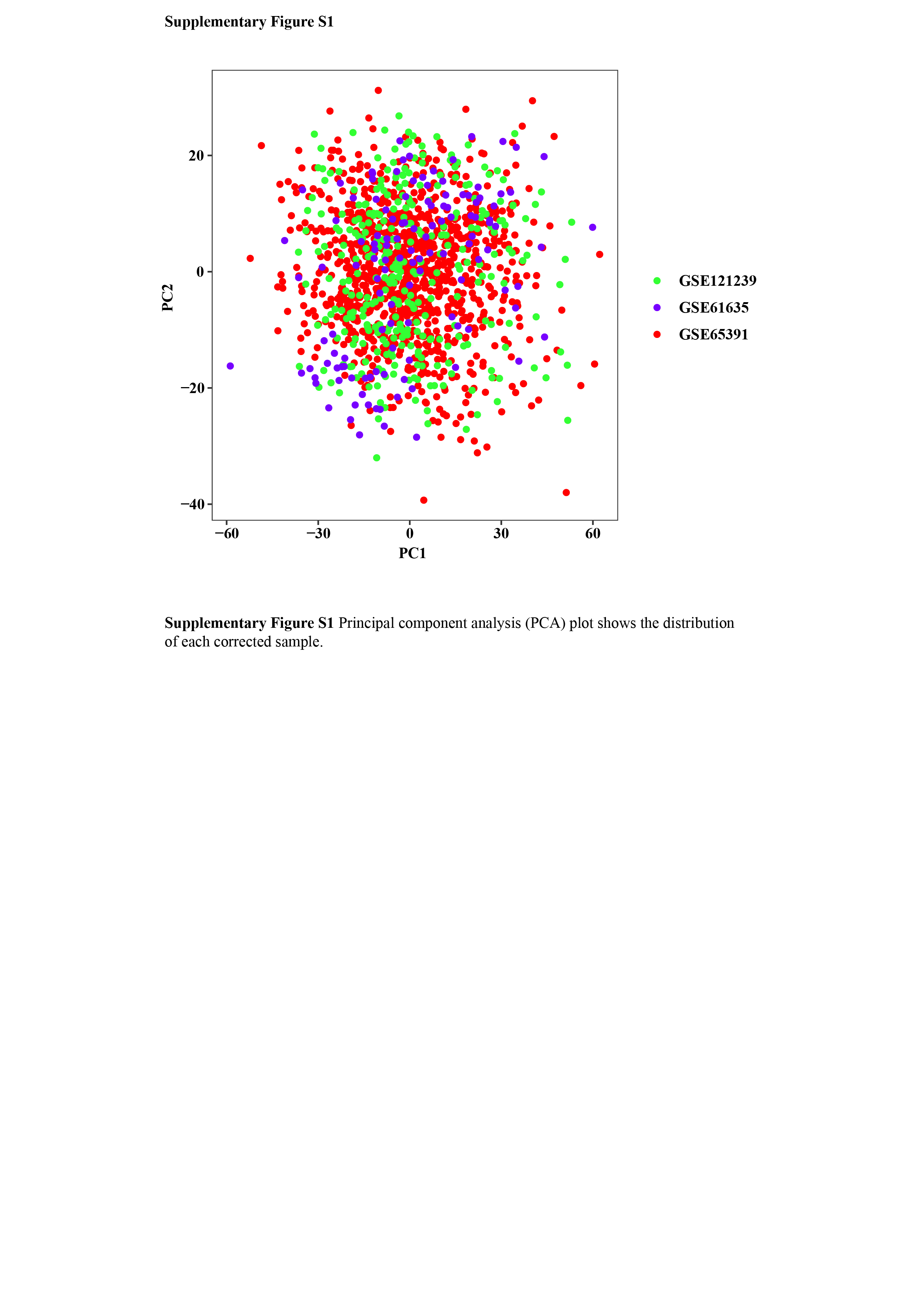

Supplement: Supplementary file 2 [file Image_1.tif]
